# Performance characteristics of a low-cost, field-deployable miniature CCD spectrometer

**DOI:** 10.1155/S1463924600000134

**Published:** 2000

**Authors:** Simon Coles, Malcolm Nimmo, Paul J. Worsfold

**Affiliations:** Department of Environmental Sciences Plymouth Environmental Research Centre University of Plymouth Drake Circus, Devon Plymouth PL4 8AA UK

## Abstract

Miniature spectrometers incorporating array detectors are becoming a viable, low-cost option for field and process deployments. The performance characteristics of one such instrument are reported and compared with those of a conventional benchtop instrument. The parameters investigated were wavelength repeatability, photometric linearity, instrumental noise (photometric precision) and instrumental drift.


Journal of Automated Methods & Management in Chemistry, Vol. 22, No. 4 (July–August 2000) pp. 97–102

### Performance characteristics of a low-cost, field-deployable miniature CCD spectrometer

**Simon Coles, Malcolm Nimmo and Paul J. Worsfold**

*Department of Environmental Sciences, Plymouth Environmental Research Centre, University of Plymouth, Drake Circus, Plymouth, Devon PL4 8AA, UK*

*Miniature spectrometers incorporating array detectors are becoming a viable, low-cost option for field and process deployments. The performance characteristics of one such instrument are reported and compared with those of a conventional benchtop instrument. The parameters investigated were wavelength repeatability, photometric linearity, instrumental noise (photometric precision) and instrumental drift.*

### Introduction

Recent advances in low-cost, miniaturized detection technology [1–3] have made it possible to take traditional laboratory-based instrumental techniques, e.g. UV/visible spectrophotometry, to remote sites for *in situ* monitoring. In recent years, field-deployable spectrophotometers have utilized light-emitting diodes as wavelength-selective sources and photodiodes as integrating detectors. However, relatively cheap miniature charge-coupled device (CCD) array detectors are now commercially available and can potentially be deployed remotely in conjunction with low-power (12 V) continuum light sources. These detection systems provide full spectrum information as opposed to discrete (or narrow band) wavelength information. They can be coupled with automated sample presentation and treatment techniques, e.g. flow injection analysis, to provide powerful, fully automated and portable analytical instrumentation for monitoring environmental and industrial processes [4, 5]. They can also potentially be used in conjunction with chemometric techniques for multi-component analysis.

In order to be able to use this technology for remote, long-term deployment, however, it is necessary to assess the analytical performance in terms of key instrumental parameters. This paper therefore reports an evaluation of a miniature CCD spectrometer (Ocean Optics PSD-1000) in terms of wavelength repeatability, photometric linearity, instrumental noise (photometric precision) and instrumental drift, and compares the results with data from a benchtop UV/visible diode array spectrophotometer.

### Experimental

### *Reagents and standards*

Solid potassium permanganate ($KMnO_4$, SpectrasoL grade, Merck, Poole, Dorset, UK) was dried for 1 h at 105 °C. A stock solution with an absorbance value of 3 a.u. was prepared by dissolving 0.2034 g of potassium permanganate in 1 l of Milli-Q water. A set of absorbance standards (0.5, 1.0, 1.5, 2.0, 2.5 a.u.) was prepared by serial dilution of the 3 a.u. stock solution. These standards were analysed immediately after preparation.

### *Instrumentation*

The portable spectrometer used in this work was a miniature fibre optic spectrometer (Ocean Optics PSD-1000, Anglia Instruments, Cambridge, UK). It consisted of two 1024-element linear CCD arrays (NEC µPD3575D) and two detector channels, designated Master (covering the UV/visible region) and Slave (covering the visible/NIR region). Figure 1 shows a schematic diagram of the spectrometer optical bench (single- not dual-channel spectrometer shown). The master channel was fitted with a collimating lens (cylin-

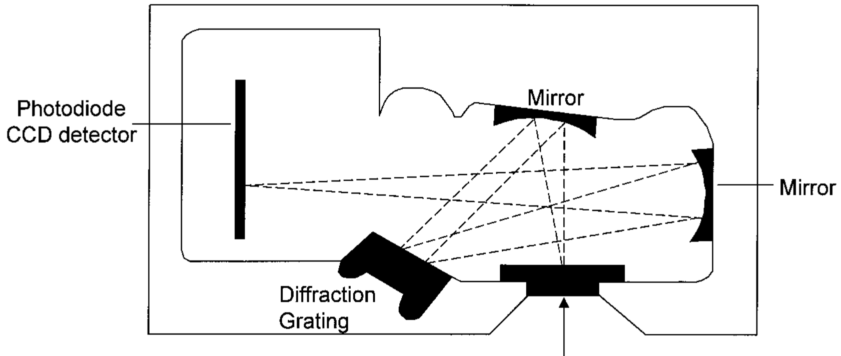

*Figure 1. Schematic diagram of the single-channel PS-1000 Ocean Optics Miniature Spectrometer optical bench.*

*Table 1. Specifications of the miniature tungsten halogen light source.*

| *Parameter* | *Specification* |
|---|---|
| Spectral range | 360–1000 nm |
| Size | 9.0 × 5.0 × 3.2 cm (LWH) |
| Weight | 185 g |
| Power input | 12 V, 750 mA |
| Power output | 6.5 watts |
| Bulb life | 900 h (standard)<br>10 000 h (long-life) |
| Bulb colour temperature | 900-h bulb = 3100 K<br>10 000-h bulb = 2800 K |
| Bulb output | 7400 foot candles |
| Output regulation | 0.2% voltage |
| Thermal stabilization | 30–40 min for 100% |
| Filter slot | 3 mm |
| Focal distance | Approx. 12.5 mm, adjustable |
| Connector | SMA 905 |

drical quartz collection lens made of Suprasil 2) to improve sensitivity. The slave channel was fitted with both a collimating lens and a 2 mm optical glass (Schott OG 515) cut-off filter to remove all radiation of wavelengths less than 515 nm.

The master grating had 600 lines $mm^{-1}$ blazed at 300 nm and enabled a spectral range of 200–700 nm. The slave grating had 600 lines $mm^{-1}$ blazed at 750 nm giving a spectral range of 500–1000 nm. The optical assembly was arranged in a Czerny–Turner configuration, with two spherical mirrors and a flat holographic grating working with first-order refracted light. All-silica core, fluorine-doped silica cladding, 200 μm single core UV/visible fibres (Anglia Instruments) were used in all applications, unless otherwise stated. A miniature tungsten halogen light source (LS-1, Ocean Optics, Orlando, FL, USA) was used as the light source (specifications given in table 1) for all experiments using the miniature spectrometer, unless otherwise stated. The light source was fitted with a 10 000 h long-life bulb which had a 2800 K colour temperature and exhibited a typical blackbody spectral response which varied slightly according to spectrometer configuration, sampling optics used and fluctuations in the power supply.

Data communication to and from the spectrometer was performed using a data acquisition card (type II PCMCIA DAQ-700, National Instruments, Newbury, Berks, UK) and a notebook PC (486-75 T2130CS, Toshiba Information Systems, Weybridge, Surrey, UK). All data acquisition was performed using Spectrascope version 2.3 (Ocean Optics) with the later exporting to Excel 5.0 (Microsoft) and Origin 2.78 (Microcal Software) for processing. Wavelength calibration was performed using a low-pressure mercury–argon calibration lamp (HG-1, Anglia Instruments).

A liquid nitrogen-cooled (140 K) two-dimensional CCD array (256 × 1024 pixels, 27.6 × 7 mm grid of 27 micron square pixels) with an imaging spectrograph (270M, Instruments S.A.) and Spectramax version 1.1d software (Jobin Yvon Optics and Spectroscopy) was used to provide a reference spectrum of the mercury–argon calibrating lamp.

A UV/visible diode array spectrophotometer (Hewlett Packard 8453) running under UV/visible Chemstation software (Hewlett Packard rev. A 02/04/95) was used as a reference benchtop spectrophotometer. The radiation source was a combination of a deuterium-discharge lamp for the ultraviolet (190–800 nm), and a low-noise tungsten lamp for the visible and short wavelength near-infrared (SWNIR, 370–1100 nm).

*Procedures*

*Wavelength repeatability*

The miniature lamp was allowed to warm up for 10 min before use and was connected to the appropriate spectrometer channel using a 50 μm UV/visible optical fibre for the master channel and a 50 μm visible/NIR optical fibre for the slave channel.

Using a data cursor function within the Spectrascope software, individual spectral lines were highlighted at their point of maximum intensity, and their reported wavelengths and pixel number recorded. Each peak was then manually identified by its reported wavelength and comparison of its peak height with neighbouring peak heights from the mercury–argon reference line spectrum. Using the 'Auto Calibration' facility within the Spectrascope software, each peak was assigned its true wavelength value. The software then performed a subpixel calibration routine [6] in order to determine the wavelength calibration coefficients for the spectrometer. The relationship between pixel number of the detector elements and wavelength is given by a second-order polynomial, $\lambda_p = I + C_1 p = C_2 p^2$, where $\lambda$ is the wavelength of pixel $p$, $I$ is the intercept corresponding to the wavelength at detector pixel 0, $C_1$ is the first coefficient and corresponds to the dispersion (nm/pixels), and $C_2$ is the second coefficient (nm/pixel$^2$) and corrects for non-linearity of response across the wavelength range.

The calibration coefficients were used to report the true wavelengths associated with each pixel and for labelling graphs and the data cursor readout within the software. These values were then entered into the 'Unit cal data' option of the software, which allowed the setting of wavelength calibration coefficients to recalibrate the spectrometer. Calibrations of both the master and slave channels of the spectrometer were performed using the calibration facility within the Spectrascope software described above. In order to assess how often recalibration of the spectrometer was required, wavelength repeatability was checked daily for 5 weeks and the individual deviations of the recorded lines from the true mercury–argon wavelengths were recorded. These individual line deviations were averaged to give an overall deviation of the whole line spectrum from the initial calibration.

*Photometric linearity*

Five spectra of the $KMnO_4$ standards were acquired (1 $s^{-1}$) using the miniature spectrometer. These readings were then averaged to give a mean absorbance value. All standards were also analysed five times using the benchtop diode array spectrophotometer and a 1-cm path length quartz cuvette.

*Instrumental noise*

Spectra of the potassium permanganate standards were acquired on the miniature spectrometer as a pseudo-continuous 15-min scan at the wavelength maximum (523 nm) using the 'History Channel'. This is a function within the software that allows user-defined operations, e.g. single-diode monitoring, mathematical functions on single diodes or a number of diodes. Up to four user-defined history channels can run simultaneously, and the history channel can be described as a digital chart recorder. Due to the need for computer processing time (dependent on the mathematical operations required), the minimum acquisition time between data points was 0.25 s, which meant that 3600 data points were acquired and averaged during the 15-min scan and exported to a spreadsheet. Instrumental noise was recorded as the peak-to-peak variation, defined as (max signal—min signal) over the 15-min scan for each absorbance standard.

*Instrumental drift*

Using the history channel, instrumental drift was assessed by monitoring a 1.0 a.u. permanganate standard at $\lambda_{max}$ over a period of 6 h. As a reference, the permanganate standard was also monitored using the benchtop diode array spectrophotometer for 6 h (21 600 s) with individual spectra recorded every 120 s, resulting in the acquisition of 181 spectra.

### Results and discussion

*Optical resolution*

The optical resolution of the miniature spectrometer [7] was defined as the dispersion (nm/pixel) multiplied by the number of pixels, where dispersion (nm/pixel) is the spectral range of the grating divided by the number of detector elements. Dispersion for the master and slave channels was 0.49 nm/pixel, and the optical resolution was 12 nm for the 200 μm fibre, 6 nm for the 100 μm fibre and 3 nm for the 50 μm fibre. The spectrometer as configured was therefore unable to resolve the pair of mercury lines at 576.960 and 579.066 nm [8]. Resolution can be improved (at the expense of spectral range and light throughput) by increasing the groove density of the grating, decreasing the slit width or optical fibre diameter, or decreasing the size of the diodes within the array. In practice, however, the majority of UV/visible spectrophotometry applications involve broad band spectra and resolution is not an important issue. Therefore, gratings were chosen to facilitate a wide spectral range (500 nm), and relatively large diameter optical fibres (200 μm) were used for increased light throughput.

*Wavelength repeatability*

Wavelength repeatability is the ability of an instrument to correctly return to the set wavelength repeatedly. It is different to wavelength accuracy, which is only of importance if measurements made on different instruments are to be compared. Conventional mechanical scanning spectrophotometers have an inherent wavelength repeatability error due to their mechanical working parts and this error increases with time as parts wear. Array instruments have no mechanical parts (except a shutter in some cases) and therefore there is negligible mechanical error. The availability of data from all parts of the spectrum, unaffected by wavelength repeatability errors, permits the freedom to chose optimum wavelengths for improved dynamic range, sensitivity and selectivity, and the increased information available can also provide the basis for other data manipulation, e.g. wavelength averaging. In addition, previously measured spectra which have been stored in computer memory can reliably be recalled for later use, aiding field deployment and negating problems associated with expensive or unstable standards.

Figure 2 shows the deviation (nm) of the master channel from the initial laboratory calibration over a period of 5 weeks. There was a net negative drift from the original calibration of −0.6 nm with a maximum deviation of −1.3 nm. Figure 3 shows a net positive deviation of 0.7 nm and a maximum deviation of 1.2 nm for the slave channel over the same period. There was no correlation between wavelength deviation and ambient air temperature. A change in absorbance of <5% due to wavelength drift during a remote deployment period can be considered acceptable for most environmental and process applications. The maximum wavelength drift recorded over a 5-week period was typically $< \pm 1.5$ nm, and therefore the effect on absorbance measurements was well within the required ±5% tolerance. With this information and consideration of field conditions compared to laboratory conditions, a field deployment of up to 5 weeks without recalibration would therefore be feasible in terms of wavelength drift. In a field environment, however, there will be significant diurnal and seasonal temperature fluctuations that will not only affect wavelength drift but other system parameters, including reaction rate. Thermostating of the detector (and other components and reagents) is therefore recommended.

*Photometric linearity*

Potassium dichromate in 0.005 M sulphuric acid is a commonly used solution standard for the routine calibration of spectrophotometers [9] and is readily available from commercial suppliers as a calibration set. However, these are used in the 200–400 nm region and therefore require a UV source. Potassium permanganate was therefore chosen as the standard for the visible region due to its well-defined absorption spectrum [10].

Figure 4 shows the overlaid spectra for permanganate standards in the range 0.0–2.0 absorbance units (at $\lambda_{max}$) recorded using the miniature spectrometer, and table 2 gives the photometric accuracy of the results. The absorbance profiles lacked the resolution of the benchtop instrument and exhibited a higher degree of noise, particularly at higher absorbances (see below). The miniature spectrometer data showed a linear correlation with predicted absorbance values ($r = 0.9999$, $n = 4$) and

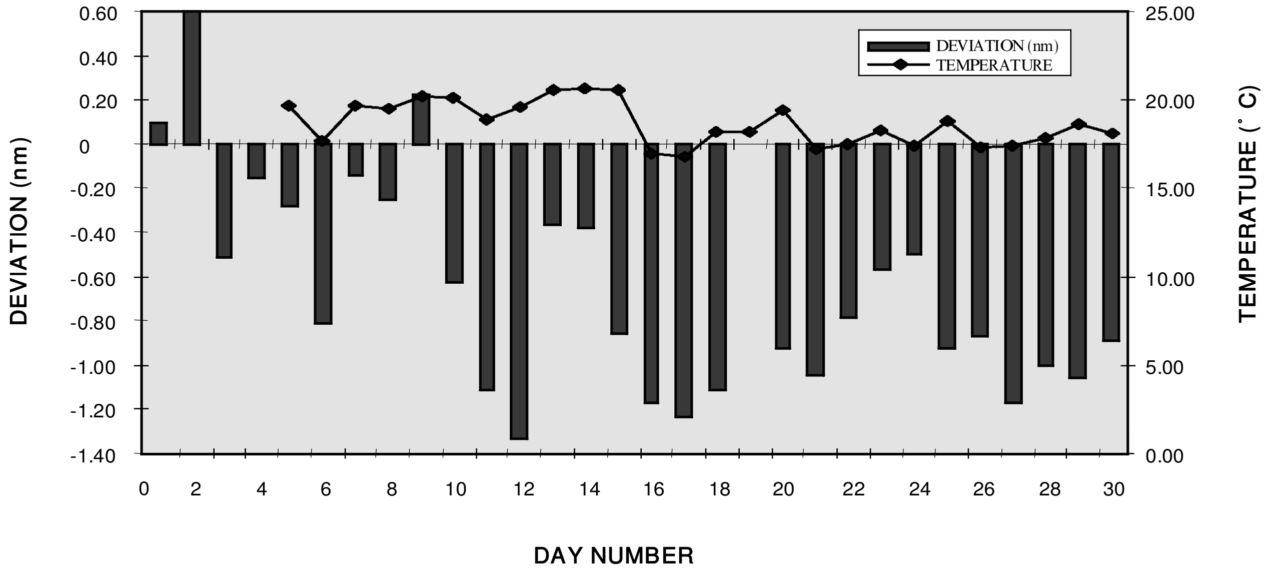

*Figure 2. Wavelength repeatability of the Master channel.*

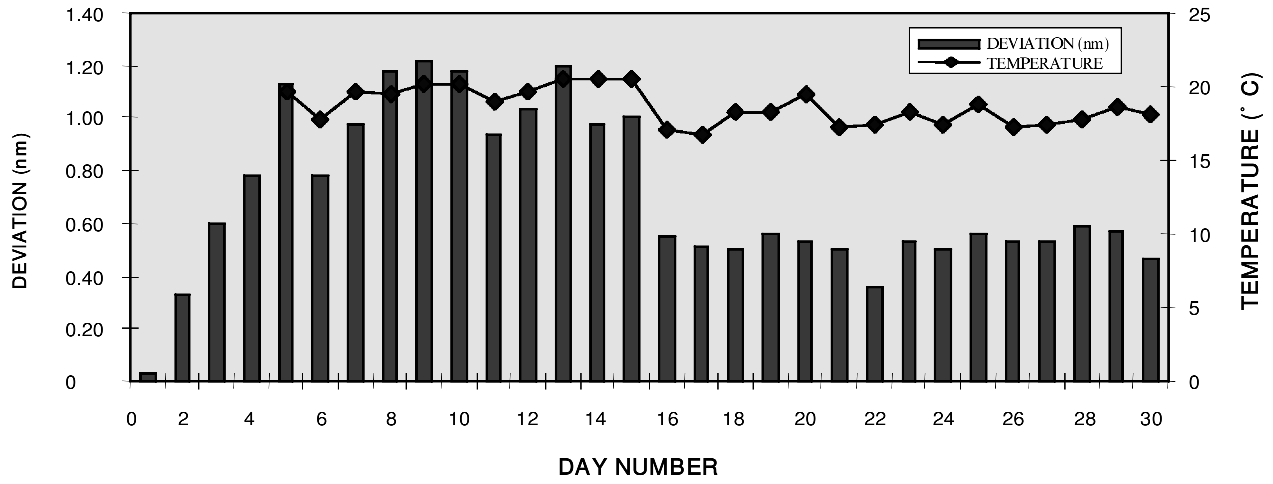

*Figure 3. Wavelength repeatability of the Slave channel.*

good agreement at lower absorbances (0.0–1.0 a.u.), but deviated increasingly from predicted values at higher absorbances. At 2.5 a.u. there was insufficient incident radiation to perform an integration and the photometric range of the instrument was therefore exceeded. This was indicative of the poor performance of the miniature spectrometer at low light levels.

*Instrumental noise (photometric precision)*

Figure 5 shows the peak-to-peak noise of potassium permanganate standards of 0.5, 1.0, 1.5 and 2.0 a.u. measured over 15-min continuous sampling periods. This clearly shows that the working range of the miniature spectrometer is 0.5–1.0 or 1.5 a.u., depending on the precision required. Such an exponential relationship is often termed case 1 noise [11], which is typical of inexpensive spectrophotometers, and is a function of the quality of the optical and electronic components used.

*Instrumental drift*

Drift is defined as long-term (hours) variation in the measured absorbance. Drift should be very low with double- or split-beam spectrophotometers because they inherently correct for this problem. However, for single-beam instruments, e.g. the miniature spectrometer, the drift is important because it is an indicator of how frequently a blank measurement must be made to ensure a desired accuracy. Table 3 shows the results obtained with the miniature spectrometer using a potassium permanganate standard of 1.0 a.u. monitored at $\lambda_{max}$ over a period of 6 h. The sampling period was divided into 26 time slices and a mean absorbance was calculated for each time slice. Maximum and minimum absorbances during a time slice, and positive and negative deviations from the mean absorbance are also reported. The large positive deviations at time slices 171–184.5 and 279–292.5 min were due to transient noise spikes. The overall drift of 0.019 a.u. $h^{-1}$ was an order of magnitude poorer than for the benchtop instrument, and although accept-

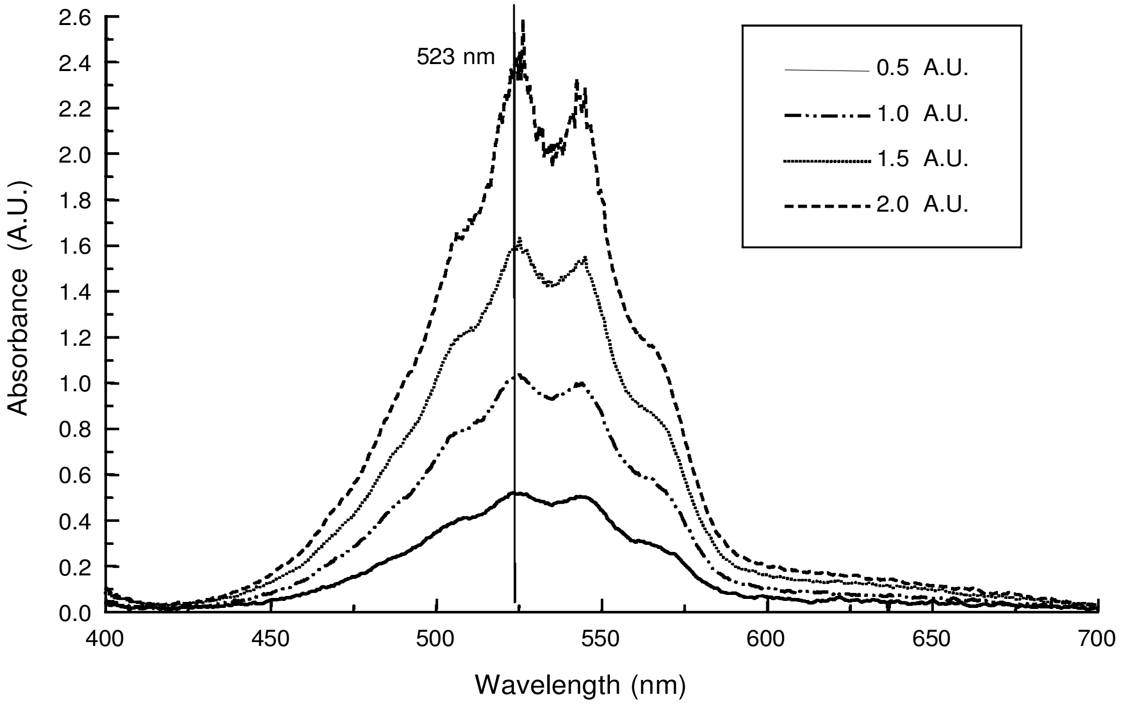

*Figure 4. Spectra of potassium permanganate standards covering the range 0.5–2.0 a.u. and acquired using the miniature spectrometer.*

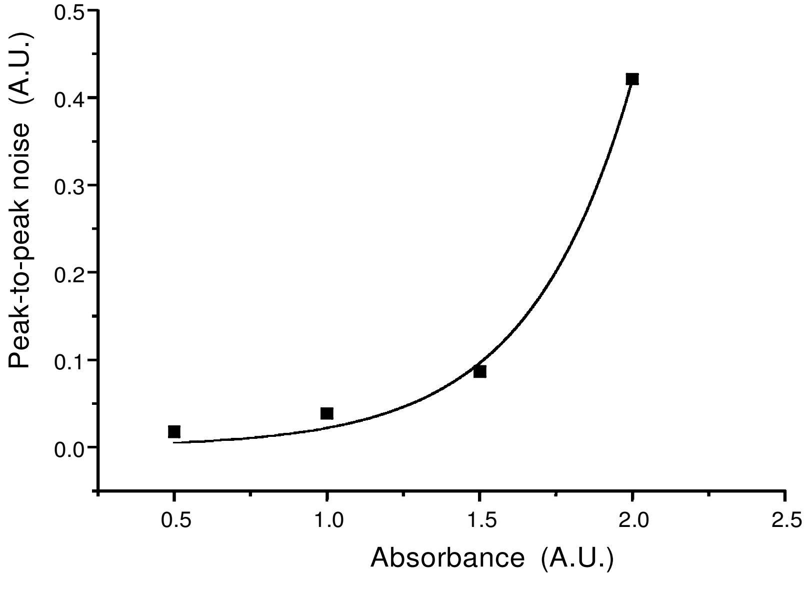

*Figure 5. Absorbance versus peak-to-peak noise for potassium permanganate standards covering the range 0.5–2.0 a.u. and acquired using the miniature spectrometer.*

*Table 2. Photometric accuracy for a range of potassium permanganate standards (five replicate spectra) acquired using the miniature spectrometer.*

| *Absolute absorbance (a.u.)* | *Miniature spectrometer (mean a.u.)* | *Error (±a.u.)* | *R.S.D. (n = 5) (%)* |
|---|---|---|---|
| 0.500 | 0.512 | +0.012 | 0.23 |
| 1.000 | 1.019 | +0.019 | 0.56 |
| 1.500 | 1.570 | +0.070 | 0.61 |
| 2.000 | 2.265 | +0.265 | 3.2 |
| 2.500 | N/A | N/A | N/A |

able for daily deployments would require periodic recalibration during longer term deployments.

### Conclusions

The miniature spectrometer is very low cost in comparison with conventional benchtop spectrophotometers, inherently portable and of rugged construction, which makes it ideally suited for field and process deployments. Wavelength repeatability, photometric linearity, instrumental noise (photometric precision) and instrumental drift are sufficiently good to allow long-term (several

*Table 3. Instrumental drift for a 1.0 a.u. potassium permanganate standard monitored at $\lambda_{max}$ for 6 h using the miniature spectrometer.*

| Time slice (min) | Mean absorvance (a.u.) | Standard deviation (a.u.) | Maximum value (a.u.) | Maximum positive deviation from mean (a.u.) | Minimum value (a.u.) | Maximum negative deviation from mean (a.u.) |
|---|---|---|---|---|---|---|
| 0–13.5 | 1.022 | 0.005 | 1.038 | 0.016 | 1.009 | 0.013 |
| 13.5–27 | 1.018 | 0.005 | 1.035 | 0.017 | 1.005 | 0.014 |
| 27–40.5 | 1.014 | 0.018 | 1.033 | 0.019 | 0.998 | 0.016 |
| 40.5–49.5 | 1.010 | 0.005 | 1.026 | 0.015 | 0.996 | 0.015 |
| 49.5–63 | 1.006 | 0.005 | 1.026 | 0.019 | 0.991 | 0.015 |
| 63–76.5 | 1.001 | 0.005 | 1.016 | 0.015 | 0.987 | 0.014 |
| 76.5–90 | 0.995 | 0.005 | 1.014 | 0.018 | 0.980 | 0.015 |
| 90–103.5 | 0.990 | 0.005 | 1.009 | 0.020 | 0.974 | 0.016 |
| 103.5–117 | 0.984 | 0.005 | 1.000 | 0.016 | 0.970 | 0.015 |
| 117–130.5 | 0.979 | 0.004 | 0.996 | 0.016 | 0.963 | 0.016 |
| 130.5–144 | 0.975 | 0.004 | 0.991 | 0.017 | 0.961 | 0.013 |
| 144–157.5 | 0.969 | 0.005 | 0.987 | 0.018 | 0.953 | 0.016 |
| 157.5–171 | 0.964 | 0.004 | 0.978 | 0.014 | 0.947 | 0.017 |
| 171–184.5 | 0.960 | 0.012 | 1.570 | 0.609 | 0.945 | 0.015 |
| 198–211.5 | 0.955 | 0.004 | 0.970 | 0.014 | 0.939 | 0.016 |
| 211.5–225 | 0.951 | 0.004 | 0.965 | 0.015 | 0.937 | 0.013 |
| 225–238.5 | 0.947 | 0.004 | 0.961 | 0.015 | 0.933 | 0.013 |
| 238.5–252 | 0.945 | 0.004 | 0.959 | 0.014 | 0.931 | 0.013 |
| 252–265.5 | 0.940 | 0.004 | 0.953 | 0.013 | 0.926 | 0.014 |
| 265.5–279 | 0.935 | 0.017 | 0.949 | 0.014 | 0.922 | 0.013 |
| 279–292.5 | 0.931 | 0.011 | 1.530 | 0.599 | 0.918 | 0.013 |
| 292.5–306 | 0.926 | 0.004 | 0.941 | 0.015 | 0.911 | 0.015 |
| 306–319.5 | 0.922 | 0.004 | 0.935 | 0.014 | 0.909 | 0.013 |
| 319.5–333 | 0.918 | 0.004 | 0.933 | 0.016 | 0.902 | 0.016 |
| 333–346.5 | 0.914 | 0.004 | 0.926 | 0.012 | 0.900 | 0.014 |
| 346.5–360 | 0.909 | 0.016 | 0.922 | 0.013 | 0.895 | 0.014 |

weeks) unattended operation (with automatic recalibration), but any application involving significant external temperature fluctuations would require a thermostated housing. Its size, optical fibre connections, rapid spectral acquisition and flexible software also make it compatible with portable flow injection technologies for monitoring transient signals.

### Acknowledgments

S. C. would like to thank the National Environment Research Council for the award of a CASE studentship and MSquared Technology for financial support.

### References

1. Osterloh, D., *Laborpraxis* , **20** (1998), 122.
2. Mazel, C. H., *Optical Engineering*, **36** (1997), 2612.
3. Belz, M., Boyle, W. J. O., Klein, K. F. and Grattan, K. T. V., *Sensors and Actuators B*, **39** (1997), 380.
4. Andrew, K. N., Worsfold, P. J. and Comber, M., *Analytica Chimica Acta*, **314** (1995), 33.
5. Clinch, J. R., Worsfold, P. J. and Sweeting, F. W., *Analytica Chimica Acta*, **214** (1988), 401.
6. Scotpatz, S., Neel, G., Romesburg, E. and Zvitz, M., *Proc. SPIE.*, **306** (1989), 1055.
7. Http://www.oceanoptics.com/specifications/Optical_Resolution.asp
8. Lide, D. R. (ed.), *75$^{th}$ Edn. CRC Handbook of Chemistry and Physics.* CRC Press, Cleveland, OH, USA (1995), pp. 10–57.
9. Burgess, C. and Knowles, A. (eds), *Standards in Absorption Spectrometry* . UV Spectrometry Group, Chapman and Hall, London (1981).
10. Thomas, M., *Ultraviolet and Visible Spectroscopy*. Analytical Chemistry by Open Learning, Wiley, Chichester (1996).
11. Skoog, D. A. and Leary, J. J., *Principles of Instrumental Analysis.* Saunders, Philadelphia (1992).

